# From the President’s desk

**DOI:** 10.4102/safp.v63i1.5362

**Published:** 2021-08-23

**Authors:** Robert Mash

**Affiliations:** 1 Division of Family Medicine and Primary Care, Faculty of Medicine and Health Sciences, Stellenbosch University, Cape Town, South Africa

By the time you read this, our National Family Practitioners Conference will be imminent, on 13–14 August, and I hope to see many of you there (https://saafp2021.org.za). Thanks to Prof. Indiran Govender and Dr Olga Maphasha who have been leading the scientific committee. Please do not miss out on this opportunity to come up to date with the latest science on COVID-19, attend continuing professional development and hear the latest family medicine research. Being fully virtual the costs are lower than usual, and of course there is no need for travel.

Since my last communication we have completed the survey of our members on their needs for continuing professional development. The survey was answered by 215 members and interestingly most were from primary care (52%) and exclusively in the private sector (49%).

In terms of practice-related issues the majority wanted help with improving the quality of their care, responding to the needs of the population served by their practice and ensuring that they practice in an ethical and professional manner. Handling telephonic consultations and the new *Protection of Personal Information Act* (POPIA) were specifically mentioned.

In terms of clinical-practice-related issues the majority wanted help with assessing and managing conditions in both adults and children, particularly diabetes. Other topics highlighted by the majority were skills in health promotion and disease prevention and emergencies in primary care. How to motivate patients towards a healthier lifestyle (e.g. tobacco smoking, healthy eating and physical activity) and wellness were specifically mentioned and how to ensure you are up to date with the latest protocols on cardiopulmonary resuscitation, particularly in children.

The South African Academy of Family Physicians (SAAFPs) is now in the process of preparing three initial short courses to respond to these learning needs. The courses will be designed and developed by Prof. Selma Smith and Dr Zelra Malan. The first two will focus on quality improvement for people with diabetes and ethico-legal issues in telehealth. I can also remind you that we already have a short on-line course on brief behaviour change counselling that meets many of the needs for skills in health promotion in the consultation (*Brief Behaviour Change Counselling* - https://healthcare-ecpd.co.za/enrol/shoppingcart/view.php?id=2939).

I am delighted to report that Dr Sheena Mathew, who is in private practice in Cape Town, has agreed to coordinate our advocacy for family physicians in the private sector. She is sending out a survey to see which of our members are in private practice and would like to be part of a private sector forum. We need to speak as a collective and understand the concerns and viewpoints of our members in private practice as we engage with the industry.

The SAAFP has also been active in commenting on the ministerial task team’s report on human resources for health in South Africa (https://saafp.org/2021/06/07/south-african-academy-of-family-physicians-response-to-the-national-department-of-health-2030-human-resources-for-health-strategy/). Although we recognise that the team had limited data in putting together their recommendations, we felt that family medicine was once again misunderstood and disregarded in the final analysis. The first model looked at the need for medical specialists, but limited itself to numbers needed in tertiary and quaternary hospitals, where it concluded that we do not need that many family physicians. The other model looked at the workforce for primary care, where it only considered the need for family physicians in district-level clinical specialist teams. The SAAFP has decided to update our national position statement on family medicine in order to communicate clearly to policymakers the contribution that we are making and can make to the health system.

Finally, we have reached a new national consensus on the learning outcomes for training family physicians, and these are published in the SA Family Practice Journal (Mash R, Steinberg H, Naidoo, M. Updated programmatic learning outcomes for the training of family physicians in South Africa. S Afr Fam Pract. 2021;63(1), a5342. https://doi.org/10.4102/safp.v63i1.5342). These new outcomes will guide training programmes in their curricula and also the College of Family Physicians in its blueprinting of the national fellowship examination in the future. We also plan to develop a set of entrustable professional activities in line with the latest directions in health professions education, which can be used in workplace-based training and assessment. The conversation on an electronic portfolio for workplace-based training continues with a group led by Prof. Louis Jenkins. The Dutch government has funded the development of such a portfolio for family medicine in South Africa, which will be piloted this year at Stellenbosch University. A long-term dream is that all programmes in the country will share one electronic portfolio.

